# Seroprevalence and epidemiological correlates of *Toxoplasma gondii *infections among patients referred for hospital-based serological testing in Doha, Qatar

**DOI:** 10.1186/1756-3305-1-39

**Published:** 2008-10-20

**Authors:** Marawan A Abu-Madi, Naema Al-Molawi, Jerzy M Behnke

**Affiliations:** 1 Department of Health Science, College of Arts and Sciences, Qatar University, PO Box 2713, Doha, Qatar; 2 Department of Laboratory Medicine and Pathology, Hamad Medical Corporation, Qatar; 3 School of Biology, University of Nottinghan, University Park, Nottingham, NG7 2RD, UK

## Abstract

**Background:**

The city of Doha in Qatar has a high density of feral cats and there is a high risk of toxoplasmosis for the resident human population. No data currently exist for the prevalence of infection with *Toxoplasma gondii *in the city.

**Methods:**

We analysed the serological response to *Toxoplasma gondii *of 1625 subjects referred for routine hospital based serological tests in Doha, Qatar. Prevalence of current/recent infection was assessed through an enzyme-linked immunosorbent assay (ELISA) for the presence of specific anti-*T. gondii *IgM antibodies, and previous history of infection through IgG.

**Results:**

Overall prevalence of IgG responses was 29.8% and this did not differ between the sexes nor between the three years of the study although there was a marked age effect. Among children less than 1 year old prevalence was 22.9%, but then dropped to <4% in the 1 year old group, indicating that these antibodies were most likely acquired *in utero *from immune mothers. Prevalence then increased steadily to peak at 41.2% among the oldest age class (>45 years). The prevalence of IgG antibody also varied significantly with region of origin, with higher rates for subjects from Africa, followed by those from the Eastern Mediterranean or Asia and lowest rates for subjects from the Arabian Peninsula. No IgM antibodies were detected in any subjects younger than 19 years, but prevalence increased to plateau at 7 – 9% in subjects aged over 20 years, and also varied with region of origin. In this case prevalence was highest among subjects from the Arabian Peninsula and least among those from Asia. Prevalence of IgM was higher among male subjects but did not vary between the three years of the study.

**Conclusion:**

Although these data are based on a selected subset of the population, they nevertheless provide the first evidence that toxoplasmosis is endemic in Qatar in the human population, and that both age and region of origin play a role in the epidemiology of the infection. Concerns relating to the role of high density of feral cats in sustaining the infection were highlighted.

## Background

Toxoplasmosis is a cosmopolitan disease arising from infection with the cat-borne Apicomplexan, coccidian protozoan *Toxoplasma gondii*, an obligate intracellular parasite that forms cysts in mammalian tissues throughout the body [1].

*T. gondii *has a worldwide distribution in human populations infecting up to one third of the global population and a wide range of other mammalian and avian species [2-7]. Toxoplasmosis is a major public health problem, with a high socioeconomic impact in terms of human suffering including the cost of caring for sick, mentally retarded and blind children [8]. The parasite is an extremely successful pathogen, responsible for significant morbidity and mortality, especially in congenitally infected and immuno-compromised individuals [4,9-11], although some subjects experience infection without overt disease or with mild symptoms [12,13]. On farms, *T. gondii *is a major cause of abortion and problems with fertility in livestock, especially among ewes [14], and therefore a significant cause of lost profitability in livestock agriculture [15-17].

The most important channels for transmission to humans are by ingestion of food or water contaminated with oocysts shed by cats, by eating undercooked or raw meat containing infective tissue cysts and via transplacental transfer, notably when the mother becomes infected for the first time during pregnancy [1,3,4,6,13,18-20]. Human infection with *T. gondii *is a huge challenge for which there is no effective treatment.

Toxoplasmosis is a zoonosis arising from man's close contact with domestic cats (*Felis catus*) [21,22]. Both domestic and wild felids are the only known definitive hosts of *T. gondii *in which the sexual cycle can take place [23], and hence cats play a central role in the epidemiology of *T. gondii*, constituting the only known source of environmental contamination with the infective oocyst stage [2]. A high risk is thus imposed on human communities that come into contact with cats [6].

As in many cities throughout the world, Doha in Qatar has had a significant rodent problem for decades and it was the difficulties in controlling these rodents that led to a solution based on the introduction of cats in the 1960s, but without any consideration of the possible knock-on effects for human health. Introduced cats have multiplied and colonized rapidly around food and water resources, mainly in urban but also in rural areas. They are known to harbour a range of helminths [24,25] and most likely also protozoa and other infectious organisms, although no surveys of the latter have been reported as yet. Cats are only rarely kept as pets in Qatar, and yet the feline population is believed to exceed 2 million, the vast majority living on the streets, scavenging garbage as well as feeding on the rodents. In the city of Doha and its surroundings, these cats mostly have a feral existence congregating near human dwellings, businesses, restaurants and in the market places where food for human consumption is prepared and traded. Because of the numbers involved Doha city has introduced a control program, but there are no plans to eliminate cats since they are believed to play a vital role in keeping down rodents populations.

This unusual high density of cats in Doha presents an obvious risk of infection to the human population and toxoplasmosis would be expected [6]. However, despite the acknowledged importance of *T. gondii*, surprisingly no report has been published yet on the incidence of toxoplasmosis in man or its prevalence in cats in Qatar. In this paper we report for the first time the prevalence of antibodies to *T. gondii *in the sera of a subset of the human population in Doha, subjects referred for serological testing in hospital. We analysed the overall prevalence of antibodies and also prevalence in relation to the available demographic variables of the subjects, including their ethnic origin, because the population of Qatar is extremely cosmopolitan with large numbers of immigrant workers from Asia, Africa, and other Arabian Peninsula and Eastern Mediterranean countries

## Methods

### Study location, selection of subjects and inclusion criteria

Doha is the capital city of Qatar, located in the Persian Gulf, having a population of about 1 million in 2007 and consisting of administrative, commercial, industrial and residential areas, with patches of agricultural land that are mostly grass fields.

The subjects for this study were 1625 individuals who had been referred for the routine TORCH series of serological/microbiological tests (*Toxoplasma*, *Rubella*, Cytomegalovirus and *Herpes*), because they had been diagnosed with one or more of the following: recent spontaneous abortion, ocular infection, liver cirrhosis, jaundice, hepato-splenomegaly. Mothers with a previous history of any one or more of these conditions were also tested routinely during pregnancy, and their most recent child usually within a year of birth. We used data that had been collected in hospitals in the period 2005–2007.

### Sample collection

Blood samples were obtained from subjects referred for examination in out-patients clinics by medically trained staff. Confidentiality was maintained throughout and the identity of subjects was not available to us, other than through their reference number, and information on age, sex and ethnic origin. The subjects were referred to the phlebotomy unit for whole blood collection (5 ml) by venipuncture in plain tubes. The blood samples were then transported to the Virology laboratory at Hamad Medical Corporation according to hospital arrangements. Blood samples were centrifuged to remove blood cells and stored at +4°C for 48 hours or frozen at -20°C for longer storage. The study was approved by Medical Research Center & Research Committee at Hamad Medical Corporation, Qatar (Research protocol # 8036/08).

### Serological testing for toxoplasmosis

*T. gondii *infections in humans can only be detected by antibody levels and the current analysis is based on the prevalence of *T. gondii *specific IgG (evidence of earlier infection, peaking at 4 months after infection and persisting at low levels for life) and on *T. gondii *specific IgM (evidence of current infection, appearing within 1–2 weeks of infection and subsiding by 6–9 months[7]).

#### IgG

We used the commercially available Enzygnost*Toxoplasmosis/IgG kit from Dade Behring Inc. USA, and followed the manufacturer's instructions. Briefly, all samples, including the *T. gondii *positive (P/N) and negative (N/N) reference samples were first diluted in a ratio of 1:20 using the coloured sample buffer POD (0.3 mol/L Tris/HCl buffer solution) and mixed well. Samples were then added to the Enzygnost* Toxoplasmosis/IgG test plates together with both positive and negative controls as per instructions. The plates were sealed with foil and incubated at 37°C for 60 mins. Then all the wells were washed four times with the diluted washing solution POD and after completion of the wash cycles, the wells were filled with 100 μl of a 1:50 dilution of anti-human-IgG/POD (enzyme conjugated- Fab fragment of rabbit antibody to human IgG in Conjugate Buffer Microbiol (37 mg of EDTA in a litre of 0.01 mol/L phosphate buffer solution). The plates were sealed with foil and placed immediately into the incubator (37°C for 60 mins.). Next the wells were washed 4 times with diluted washing solution POD (1:20 dilution of Washing Solution POD (Phosphate buffered) with distilled water). After completing the wash cycles, the wells were filled with 100 μl of working chromogen solution (1 ml of Chromogen TMP (tetramethylbenzidine dihydrochloride) with 10 ml of buffer/Substrate TMB (hydrogen peroxide) and sealed with fresh foil. The plate was incubated immediately at room temperature (15–25°C) for 30 mins, protected from light. Finally 100 μl of stopping solution POD were added to each well and kept at room temperature for 30 mins. Results were quantified in the photometer at 450 nm within 1 hour.

#### IgM

IgM antibody levels were quantified using the commercially available Enzygnost*Toxoplasmosis/IgM assay (Dade Behring, U.S.A), and by following the manufacturer's instructions. Essentially the assay was conducted as for IgG except that a goat antibody to human IgM was used to detect IgM bound to the plates.

### Preparation of data for analysis

All birth dates and dates of examination were recorded meticulously, and the ages of subjects were calculated to the nearest month (for those below 3 years of age) and to the nearest year for those older. Seven age classes were then constructed but these did not span comparable age ranges because we wished to scrutinise particularly carefully prevalence of antibody in children less than one year of age and those from 12 to 24 months old. Other classes were constructed to span young children (2–10 years) and teenagers (11–20 years), and then young (21–29 years), middle aged (30–45 years) and older (>45 years) subjects in the sample.

The subjects or their parents (in the case of young children) in this study came from 41 countries. For the purpose of analysis these were grouped into 4 regions. These were as follows (in parenthesis we give the percentage of total sample for each country of origin, if more than 0.1%):

Arabian Peninsula (Bahrain (0.7%), Kuwait, Oman (0.7%), Qatar (32.4%), Saudi Arabia (1.4%), United Arab Emirates (0.4%) and Yemen (3.1%)).

Africa (Algeria (0.3%), Egypt (11.7%), Eritrea (0.1%), Ethiopia (0.4%), Kenya, Libya (0.1%), Mauritania (0.25%), Morocco (0.5%), Senegal, Somalia (0.7%), S. Africa, Sudan (3.3%), Tunisia (1.0%)).

Eastern Mediterranean area (Jordan (5.5%), Lebanon (1.0%), Palestine (9.8%), Syria (3.0%), Turkey (0.2%)).

Asia (Afghanistan, Bangladesh (1.4%), India (11.3%), Indonesia (1.5%), Iran (1.4%), Iraq (1.3%), Macao, Nepal (2.2%), N. and S. Korea, Malaysia, Pakistan, Philippines (3.0%), Sri Lanka (1.4%), Thailand, Vietnam).

### Statistical analysis

On the basis of the ELISA, subjects were diagnosed as either positive/negative for specific IgG and IgM antibodies to *T. gondii*. Prevalence data (percentage of subjects showing a positive ELISA) are shown with 95% confidence limits, calculated as described by Rohlf & Sokal [26] employing bespoke software. We used a two stage method for the analysis. Firstly the data were analyzed by maximum likelihood on log linear analysis of contingency tables using the software package SPSS (Version 12.0.1.). Full factorial models incorporated age (7 age classes corresponding to : up to one year, one year, two to 10 years, 11–20, 21–29, 30–45, more than 45 years), sex (2 levels, males and females), ethnic origin (four levels Arabian Peninsula, African, Eastern Mediterranean and Asian) and year of study (3 levels, 2005, 2006, and 2007). Evidence of infection by the ELISA was considered as a binary factor (present/absent of *Toxoplasma *specific antibody). Beginning with the most complex model, involving all possible main effects and interactions, those combinations that did not contribute significantly to explaining variation in the data were eliminated in a stepwise fashion beginning with the highest-level interaction. A minimum sufficient model was then obtained, for which the likelihood ratio of *χ*^2 ^was not significant, indicating that the model was sufficient in explaining the data. The importance of each term (i.e. interactions involving presence/absence of antibody) in the final model was assessed by the probability that its exclusion would alter the model significantly and these values are given in the text.

We also fitted general linear models (GLM) with quasibinomial errors (because there was a disparity between residual degrees of freedom and residual deviance in models fitted with binomial errors) implemented in R version 2.2.1 (R Core Development Team). Full factorial models (with age class, sex, region of origin and year of study, fitted as fixed factors with levels identical to those specified above) that converged satisfactorily were simplified stepwise by deletion of terms beginning with the highest order interaction and were evaluated by comparing models with or without that interaction or main effect. Changes in deviance were interpreted with *χ*^2 ^tests. Minimum sufficient models were then fitted (all significant interactions and main effects + any main effects that featured in interactions) and the process was repeated to obtain values for changes in deviance, test statistics and probabilities.

## Results

### Demography of the study population

The study comprised 1625 subjects (1077 females and 548 males). Table 1 shows the distribution of subjects among the age classes that were constructed for the purpose of analysis of age effects. Although 338 children less than one year old were tested, the numbers of 1 year old children and 2–10 years old were very low by comparison with other age classes. Table 1 also shows the distribution of subjects by region of origin. Most subjects came from countries in the Arabian Peninsula including Qatar itself and its close neighbours, and fewest from the Eastern Mediterranean region, but most categories were sufficiently well represented to enable statistical analysis.

**Table 1:** Numbers of subjects enrolled in the study by sex and age class

	By sex		By region of origin^1^				
			
Age class^2^	Males	Females	1	2	3	4	Total
<1 yr	179	159	168	45	30	95	**338**
1 year	17	12	14	7	6	2	**29**
2–10	28	35	21	13	11	18	**63**
11–20	28	61	54	10	14	11	**89**
21–29	79	340	155	92	44	128	**419**
30–45	134	405	169	116	67	187	**539**
**>45**	**83**	**65**	**49**	**21**	**8**	**70**	**148**
							
**Totals**	**548**	**1077**	**630**	**304**	**180**	**511**	**1625**

1. Region 1 = Arabian Peninsula, 2 = Africa, 3 = Eastern Mediterranean, 4 = Asia.

2. Age range in each of the age classes used for analysis

### Prevalence of IgG antibody responses

A total of 484 subjects were found to be positive for IgG antibodies, corresponding to 29.8% of the study group. When other factors had been taken into consideration, there was no significant difference between the sexes (males = 27.6%, Cl_95 _= 24.7 – 30.6% and females = 30.9%, Cl_95 _= 31.1 – 37.0%), and no significant difference between the three years of the study (2005 = 32.2%, Cl_95 _= 29.3 – 35.4%; 2006 = 28.6%, Cl_95 _= 22.8 – 35.0%; 2007 = 28.5%, Cl_95 _= 25.5 – 31.7%).

**Figure 1:**
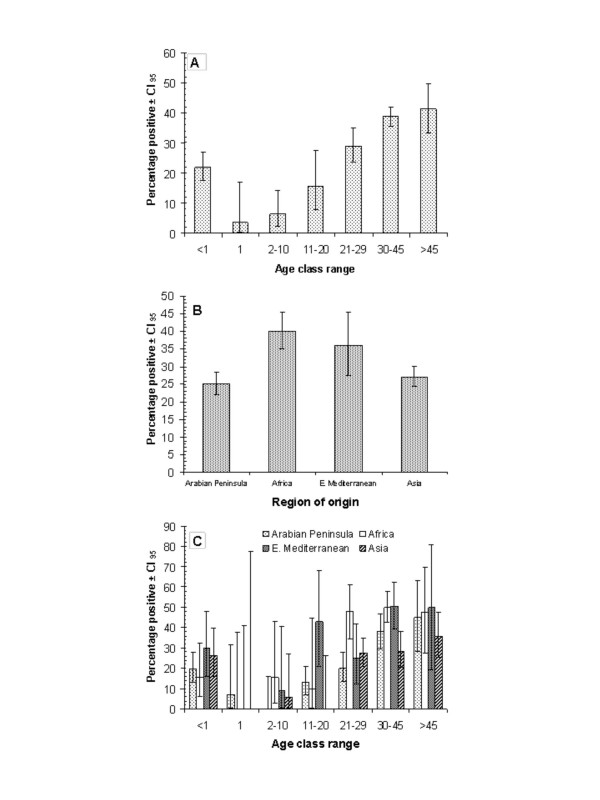
**Seroprevalence of anti *Toxoplasma gondii *IgG antibodies in subjects in Qatar**. A. By age class. The number of subject in each age class in the order as shown on the abscissa (from age class <1 through to age class >45) was 338, 29, 63, 419, 539 and 148 respectively. The total sample size was 1625. B. By region of origin. The number of subjects of each ethnic origin was as follows Arabian Peninsula = 630, Africa = 304, Eastern Mediterranean = 180 and Asia = 511. C. By age class and region of origin.

Figure 1 presents the distribution of prevalence with *T. gondii *among different age groups (1a) and among subjects from different ethnic regions in 2005–2007 (1b). Analysis revealed a significant interaction between age and region of origin (region of origin*age class* presence of IgG, *χ*^2^_18 _= 34.9, *P *= 0.01) and these data are illustrated in Fig. 1c. The prevalence of positives among the newborn children was 21.9% but then dropped markedly to the lowest values in the one year old children despite the smaller sample size of this age class (only 1 positive from 29 subjects). Prevalence then climbed steadily with each subsequent age class, peaking among the oldest subject (>45 years old) at 41.2%. When the age classes were pooled, the highest prevalence of positives was among subjects with an African origin (Fig. 1b, 40.1%) and lowest among those from Arabian Peninsula countries (Fig. 1b, 25.0%). Figure 1c shows that positives were detected in the youngest age class in all four groups, and that in all four groups positives were rare in the age classes spanning 1 to 10 years. In all four groups positives were then seen with increasing frequency as subjects aged, except for a dip in the 21–29 year age class among subjects of an Eastern Mediterranean origin. The lowest percentage of positives was seen among middle aged and older subjects from Asia. Thus, although the age-prevalence pattern was clearly evident and generally similar in all four groups, it nevertheless showed some variation between subjects of different ethnic origin.

Analysis by R, fitting a GLM with a quasibinomial error structure, confirmed the highly significant main effects of age (change in deviance = 84.45, *P *< 0.0001, for 6 df) and region (change in deviance = 27.03, *P *< 0.0001, for 3 df), and the 2-way interaction between age and region (change in deviance = 34.85, *P *= 0.01, for 18 df, residual degrees of freedom = 1615).

### Prevalence of IgM antibody responses

IgM antibody was detected less frequently than IgG with a total of 94 subjects giving a positive ELISA (5.8%). With other factors taken into consideration, there was no significant change in the prevalence of positive subjects across the three years of the study despite an apparent downward trend across the period (2005 = 7.4%, Cl_95 _= 5.8 -9.4%; 2006 = 5.6%, Cl_95 _= 3.1 – 9.6%; 2007 = 4.4%, Cl_95 _= 3.1 – 6.1%), representing a drop of 3%.

**Figure 2:**
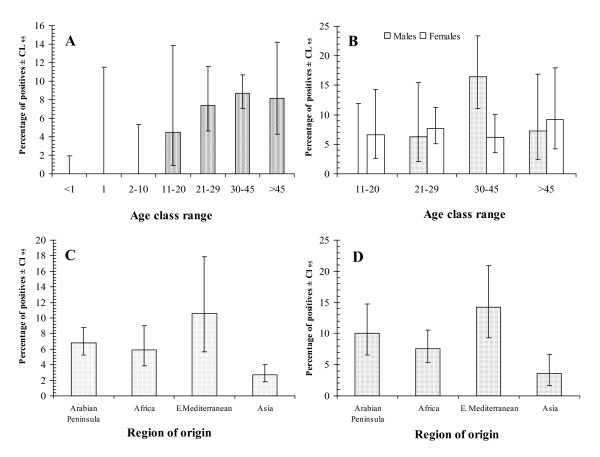
**Seroprevalence of anti *Toxoplasma gondii *IgM antibodies in subjects in Qatar**. A. By age class in the whole study population (*n *= 1625). B. By age class and host sex in the 4 older age classes (11+ years old; *n *= 1195). C. By different regions of origin in the whole dataset (*n *= 1625). D. By regions of origin but confined to the 4 older age classes (10+ years, *n *= 1195).

Figure 2 shows the epidemiological correlates of *T. gondii *infection in men and women, among different age group and from different ethnic origins. Analysis by maximum likelihood revealed a simple minimum sufficient model with just three main effects. Thus prevalence of subjects with IgM for *T. gondii*, varied independently in relation to ethnic origin (region of origin* presence of IgM, *χ*^2^_3 _= 31.1, *P *< 0.001) and age (age class* presence of IgM, *χ*^2^_6 _= 77.5, *P *< 0.001). The strongest effect was that of age. No *T. gondii *specific IgM was detected in any children in the first three age classes (>1, 1 and 2–10 years old). In fact the youngest subject with antibodies of this class was aged 19 years. Prevalence then plateaued at 7–9% among subjects over 20 years of age. Subjects from the Eastern Mediterranean region exhibited the highest prevalence of IgM (10.6%) and those from Asia the lowest (2.7%). With age class and region of origin taken into consideration, there was also a significantly higher prevalence of IgM among male subjects (sex* IgM, *χ*^2^_1 _= 10.5, *P *= 0.001), although this was not clearly reflected in the means and confidence limits extracted from the pooled data-set (males = 6.0%, Cl_95 _= 4.5 – 7.9% and females = 5.7%, Cl_95 _= 5.6 – 8.8%). However, it was clearly evident when prevalence of specific IgM was calculated for subjects older than 10 years of age (males = 10.2%, Cl_95 _= 7.1 – 14.1%; females = 7.0%, Cl_95 _= 5.0 – 9.5%), among whom all the positives were found.

Since no IgM was detected in the three youngest age classes, comprising 430 subjects, the data were re-analysed excluding these groups. This yielded a significant sex*age*presence of IgM interaction (*χ*^2^_3 _= 20.4, *P *= 0.0001) which is illustrated in Fig. 2B. In the youngest age class (11–20 years old) no males were positive, whereas prevalence was 6.6% among females. In the 30–45 year age class prevalence of specific IgM was more than twice as high in males compared with females (16.4 versus 6.2%). Figure 2D shows the prevalence of specific IgM antibodies among subjects from the four regions of origin but confined to the four older age classes, and the pattern is much the same as when the whole data-set was analyzed (Fig. 2C).

Analysis of the whole data-set by R, fitting a GLM with a quasibinomial error structure, confirmed the highly significant main effects of region of origin (change in deviance = 31.1, P < 0.0001 for 3 df), age (change in deviance = 77.5, *P *< 0.0001, for 6 df) and sex (change in deviance = 10.5, *P *< 0.0002, for 1 df), and identified also a 2-way interaction between age and sex (change in deviance = 11.9, P = 0.02, for 6 df, residual degrees of freedom = 1614).

## Discussion

The results of this study, based on serological tests, confirmed our expectation that toxoplasmosis is indeed endemic in Qatar and that a substantial proportion of the referred patients showed evidence of earlier infection (IgG), whilst fewer had evidence of current infection (IgM). Recent infections were detected only in subjects who were 19 years old and older, and stabilised at 7–9% in all the older age classes, indicating that in Doha adults of all ages were becoming infected with *T. gondii *for the first time. The rising prevalence of IgG positive subjects with increasing age also confirmed that infections were occurring in all age classes throughout adult life.

The study group comprised subjects that had been referred for the TORCH series of serological/microbiological tests as explained earlier, and for this reason they cannot be considered as a random selection of the population in the country. Nevertheless, since there are no other data in the public domain on toxoplasmosis in Qatar, we considered it worthwhile to analyse the trends in prevalence among this selected group, as a first step in a longer-term investigation of the role of feral cats in the transmission of toxoplasmosis among the human population in Qatar and the associated risks to human health.

The prevalence and risk factors for the transmission of *T. gondii *vary substantially between countries and geographic regions. Toxoplasmosis is known to be present in the Middle East with reports of infections in cats and/or people in Egypt [27,28], Jordan [29], Jerusalem [30], Lebanon [31], Saudi Arabia [32-34] and Iran [35,36]. It has been reported as particularly high among a randomly selected proportion of the population of Kuwait [37].

In this study IgG antibodies to *T. gondii *were found to be five times more common than IgM antibodies (29.8% versus 5.8% in the study group). Clearly the overall prevalence of IgG antibodies, reflecting earlier infection with *T. gondii*, among these referred subjects indicated that between 1 in 3 and 1 in 4 had experienced earlier infection, and these values are broadly in line with reports from other countries [38]. However, the changes of prevalence of IgG positive samples across age groups revealed a more complex story. Almost a quarter of children less than one year old had IgG antibodies and these were most likely acquired from their mothers by transplacental transfer [39]. By one year of age the prevalence rate had dropped to below 5%, presumably through catabolism of passively transferred antibodies, supporting the idea that higher prevalence among younger children reflected acquired maternal antibodies rather than active infection. Prevalence then increased markedly with each successive age class as reported previously in other studies [40], peaking in the older subjects at over 40%. This rising trend with age, reflects the continuing risk of infection throughout adult life and arises from the cumulative risk of exposure and infection with age in an environment where transmission is encouraged by the high density of feral cats.

Interestingly the age related trend in prevalence of IgG antibodies in the combined study group, was also seen in each of the four groups representing countries of origin of the patients referred for examination. In each regional group prevalence of IgG was higher among children less than one year of age compared to those one year old, and in each region prevalence rose with increasing age despite some of the minor perturbations that generated the statistical interaction between region of origin and age. The overall trend in each regional group was similar, indicating that there is ongoing and repeated exposure to *T. gondii *in all groups.

IgM antibodies are considered to reflect active current/recent infection [7], and not surprisingly, prevalence rates were lower. The absence of IgM antibodies in children less than 2 years of age is consistent with the observation that this class is not transferred across the placenta [39], and with the interpretation of the IgG antibodies as being derived from the maternal circulation in the youngest individuals. In fact the youngest subject with IgM antibodies was 19 years old. These current/recent infections, as reflected in the prevalence of IgM positives, plateaued at about 7–9% among the older patients, and since IgM antibody concentrations normally decline within a year of infection [7], these results indicate that the risk of infection was very similar for each age class over 21 years of age. There was just the one interesting exception among 30–45 year old males among whom active infections were detected in over 16% of subjects. We cannot offer an explanation of this finding.

Although Qataris represented the largest group in this study, they comprised only 32.4% of the subjects for this study, and were least likely to show evidence of earlier infection (data not shown). The remainder were subjects from 40 additional countries of which the next most prominent nationality were Egyptians. Subjects from African countries were more likely to show a positive IgG test, whereas active/recent infections (IgM) were more common among those of Eastern Mediterranean origin, but despite the statistically valid differences between these groups, the differences in prevalence rates were actually not marked (7% difference for IgM and 15% difference for IgG, between the group with the highest and lowest in each case). Considerably greater disparities in prevalence between different geographical regions have been reported previously by others [38]. It was also of interest that after the influence of age, sex and region of origin had been taken into account, there was no change in prevalence rates across the three years of the study, and this contrasts with reports that the prevalence of toxoplasmosis has been steadily falling in recent decades in some countries [41-43].

Although our data cannot be considered to reflect prevalence rates throughout the entire population, they provide the first quantitative data on toxoplasmosis in the human community in the country, albeit a subset of that community that was referred for testing. Despite the rather select group of subjects in this study, our data clearly show that *T. gondii *infections are not rare among the inhabitants of Doha, and are likely perpetuated by the feral cat population [6]. At this stage we cannot link the prevalence of infections to spontaneous abortions and other known consequences of infection with this parasite [12], and therefore cannot assess comprehensively the full risk of *Toxoplasma *related pathologies in the local community but we have already begun to collect relevant data to enable such analyses to be conducted in the future. Because cats are still valued for their vital role in keeping down rodent populations in Doha city, there are no current plans to eliminate them. Since the principal source of infection is not likely to be removed in the near future, people, but especially immunocompromised individuals and pregnant women, should be made aware of the routes of transmission and encouraged to observe high standards of hygiene, especially after contact with cats, soil and before meals.

To conclude, our study has emphasised the unique epidemiological situation in Qatar where cats outnumber humans by two to one. Our study confirms for the first time the presence of anti-*T. gondii *specific antibodies in human populations in Qatar. Since cats are key to the transmission of *T. gondii*, more extensive epidemiological studies are warranted to assess the extent to which feral cats actually harbour current infections and to evaluate comprehensively the risk of transmission to other vertebrates, including humans. The full picture will only emerge after further studies with a broader remit are conducted in the country, covering a wider range of inhabitants, the meat industry, and environmental contamination. Such studies have already been initiated.

## Competing interests

The authors declare that they have no competing interests.

## Authors' contributions

MAAM conceived the study, collected the data, and wrote the paper. NAM participated in the serological tests and helped in data collection. JMB analysed the data and wrote the paper.
